# The Effect of Nanosystems on ATP-Binding Cassette Transporters: Understanding the Influence of Nanosystems on Multidrug Resistance Protein-1 and P-glycoprotein

**DOI:** 10.3390/ijms21072630

**Published:** 2020-04-10

**Authors:** Francisco V.C. Mello, Gabriela N. de Moraes, Raquel C. Maia, Jennifer Kyeremateng, Surtaj Hussain Iram, Ralph Santos-Oliveira

**Affiliations:** 1 Brazilian Nuclear Energy Commission, Nuclear Engineering Institute, Rua Helio de Almeida 75, Ilha do Fundão, CEP 21941-614 Rio de Janeiro, Brazil; francisco_dovale@yahoo.com.br; 2 Laboratory of Cellular and Molecular Hemato-Oncology, Program of Molecular Hemato-Oncology, Brazilian National Cancer Institute (INCA), CEP 20230130 Rio de Janeiro, Brazil; gabrinestal@yahoo.com.br (G.N.d.M.); racmaia@inca.gov.br (R.C.M.); 3 Department of Chemistry & Biochemistry, College of Natural Sciences, South Dakota State University, Brookings, SD 57007, USA; kyeremateng.jeniffer@sdstates.edu (J.K.); surtaj.iram@sdstate.edu (S.H.I.); 4 Laboratory of Radiopharmacy and Nanoradiopharmaceuticals, Zona Oeste State University, Campo Grande, CEP 23070200 Rio de Janeiro, Brazil

**Keywords:** cancer resistance, nanosystems, P-glycoprotein, multidrug resistance associated protein-1, graphene quantum dots, mesoporous silica nanoparticles, polymeric lactic acid nanoparticles

## Abstract

The cancer multidrug resistance is involved in the failure of several treatments during cancer treatment. It is a phenomenon that has been receiving great attention in the last years due to the sheer amount of mechanisms discovered and involved in the process of resistance which hinders the effectiveness of many anti-cancer drugs. Among the mechanisms involved in the multidrug resistance, the participation of ATP-binding cassette (ABC) transporters is the main one. The ABC transporters are a group of plasma membrane and intracellular organelle proteins involved in the process of externalization of substrates from cells, which are expressed in cancer. They are involved in the clearance of intracellular metabolites as ions, hormones, lipids and other small molecules from the cell, affecting directly and indirectly drug absorption, distribution, metabolism and excretion. Other mechanisms responsible for resistance are the signaling pathways and the anti- and pro-apoptotic proteins involved in cell death by apoptosis. In this study we evaluated the influence of three nanosystem (Graphene Quantum Dots (GQDs), mesoporous silica (MSN) and poly-lactic nanoparticles (PLA)) in the main mechanism related to the cancer multidrug resistance such as the Multidrug Resistance Protein-1 and P-glycoprotein. We also evaluated this influence in a group of proteins involved in the apoptosis-related resistance including cIAP-1, XIAP, Bcl-2, BAK and Survivin proteins. Last, colonogenic and MTT (3-(4,5-dimethylthiazol-2-yl)- 2,5-diphenyltetrazolium bromide) assays have also been performed. The results showed, regardless of the concentration used, GQDs, MSN and PLA were not cytotoxic to MDA-MB-231 cells and showed no impairment in the colony formation capacity. In addition, it has been observed that P-gp membrane expression was not significantly altered by any of the three nanomaterials. The results suggest that GQDs nanoparticles would be suitable for the delivery of other multidrug resistance protein 1 (MRP1) substrate drugs that bind to the transporter at the same binding pocket, while MSN can strongly inhibit doxorubicin efflux by MRP1. On the other hand, PLA showed moderate inhibition of doxorubicin efflux by MRP1 suggesting that this nanomaterial can also be useful to treat MDR (Multidrug resistance) due to MRP1 overexpression.

## 1. Introduction

### 1.1. Cancer Today

Cancer’s incidence and mortality are increasing globally due to complex reasons which include but are not limited to population growth and aging. Socioeconomic status of different regions which affects cancer diversity on men and women plays an important role as well [1]. Speaking of which, cause of deaths can change drastically across regions. However, cancer reached the first and second leading cause of death before the age of 70 years in countries such as The United States and Brazil, respectively, and many others [1], and it is the second leading cause of death worldwide which is concerning [2,3].

When we look closer to cancer, incidence and mortality are influenced by many variables such as risk factors and lifestyle, cancer type and its heterogeneity, screening and imaging tools at disposal, early or late diagnosis, region of incidence and prevention and health programs available locally and the provision of an efficient and efficacy treatment. Many of these variables should be considered in the adoption of the best chemotherapy for patients [1]. Unfortunately, the efficiency of treatment regimens to a wide range of neoplasms can highly vary and many authors argue that changes in the development of new treatments should be considered [4,5].

An important player involved in the failure of treatment in cancer is the resistance against chemotherapy known as multidrug resistance (MDR). It is a phenomenon that has been receiving great attention in the last 50 years [6,7] due to the sheer amount of mechanisms discovered and involved in the process of resistance which hinders the effectiveness of many anti-cancer drugs [8,9,10,11,12,13]. It is a challenge that needs to be further investigated and overcome and is a paramount factor in cancer recurrence and low survival rates [14,15,16,17]

### 1.2. Multidrug Resistance in Cancer

Among the mechanisms responsible for MDR, the ATP-binding cassette (ABC) transporters are a group of plasma membrane and intracellular organelle proteins involved in the process of externalization of substrates from cells, and these proteins can be highly expressed in cancer [18,19]. They are usually composed of a functional unit made of two transmembrane domains (TMD) and two nucleotide ATP-binding domains (NBD). In humans, they are a family of 48 genes expressing ATP-dependent proteins, some of them are responsible for the active efflux of xenobiotics such as anti-cancer drugs [20,21,22].

ABC proteins are involved in several physiological roles in the organism related to the clearance of intracellular metabolites, ions, hormones, lipids and other small molecules. These roles can directly affect drug absorption, distribution, metabolism and excretion in tissues [20,21,23]. In addition, ABC proteins can be found at important pharmacological barriers such as in the blood-brain-barrier (BBB) protecting the tissue from exogenic solutes [12]. Furthermore, ABC drug transporters can be responsible for a specific role in the efflux of different kind of substrates (e.g., hydrophobic or hydrophilic substrates), but some overlap in function exists among them which makes it even a more complex matter to overcome since a new solution in treatment would need to deal with more than one transporter protein [24,25].

The first ABC transporter protein observed was the cell surface P-glycoprotein (P-gp) or subfamily ABCB1 [8,26]. It is normally found in the epithelium of the gastrointestinal tract, kidney, liver, placenta, at pharmacological barrier sites, stem cells and in the immune system [12]. It is reported that P-gp (protein and mRNA) overexpression can be found in leukemia, and colon, colorectal, breast, ovarian and lung cancers [27]. P-gp transports neutral or cationic hydrophobic and unmodified compounds, and it is involved in the resistance of several important chemotherapy agents including anthracyclines (e.g., doxorubicin), taxanes (e.g., paclitaxel), vinca alkaloids (e.g., vinblastine), antibiotics (e.g., actinomycin D), tyrosine kinase inhibitors (TKIs) (e.g., imatinib), epidermal growth factor receptor TKIs (e.g., erlotinib) which shows why this transporter can modulate the efficiency of treatment regimens used in many cancer types [28]. Lastly, glycoprotein-P can participate in the apoptosis-related resistance either by its efflux activity or by regulating cell proliferation, differentiation and death [29]. For instance, the downregulation of P-gp expression can affect the downregulation of survivin expression—an anti-apoptotic protein-demonstrating that they might share same regulators and therefore, modulate apoptosis [29,30,31].

The multidrug resistance protein 1 (MRP1) or ABCC1, first observed in doxorubicin-resistant cell lines [11,19,32,33], is involved in cancer resistance to drugs as well, usually overlapping its efflux selectivity to substrates with the ones transported by P-gp. However, unlike P-gp, MRP1 cannot transport taxanes, but can transport anionic lipophilic substrates which helps differentiate its efflux activity. In addition, MRP1 can transport modified compounds such as those that have been conjugated to glutathione, glucuronide or sulfate [34]. Lastly, it is normally found on the basolateral surface of the epithelial membrane of tissues [11,32].

### 1.3. The State of ABC Transporters Modulators

ABC transporters have been used as targets by selective modulators aiming to reduce its activity or revert the resistant phenotype in cancer, increasing the concentration of anti-neoplastic drugs in cells [22,25,28]. This rationale can be achieved by using molecules and/or monoclonal antibodies that will interact directly with the transporters extracellularly or act as an antagonist in the cytoplasm [14]. Another strategy is to use interference RNA (RNAi) to silence the expression of *ABC* genes [5].

However, the first (e.g., Verapamil, Cyclosporin, Tamoxifen, Calmodulin) and second (e.g., Dexverapamil, Valspodar, Biricodar) generation of inhibitors were not successful in trials due to nonspecificity, the need of high concentrations that lead to toxicity, alteration of the pharmacokinetics of cytotoxic drugs due to drug-drug interactions in co-administration and formulation problems (e.g., solubility, biocompatibility, stability) [14,18]. The third (e.g., Laniquidar, Elacridar, Tariquidar) and fourth generation (e.g., Neochamaejasmin B, Curcumin) of modulators are promising candidates since they show less influence in pharmacokinetics and less toxicity [35,36].

### 1.4. Apoptosis on Cancer Resistance

Apoptosis is a programmed cell death that aims to control the healthy survival and death balance in normal cells and is regulated by a variety of proteins [37,38]. Defects on apoptosis play an important role in tumor pathogenesis, enabling cancer cells to survive independently the pro-apoptotic stimuli, including the anticancer drugs activity [38].

Cell signaling pathways and proteins involved in the cell death mechanism can be responsible for cancer resistance, as well [39,40]. Anti-apoptotic proteins such as the Inhibitors of apoptosis proteins (IAP) family (NIAP, cIAP-1, cIAP-2, XIAP, Livin, BIRC6, ML-IAP, ILP2, Survivin) and Bcl-2 family can be overexpressed in cancer cells, and pro-apoptotic proteins such as BAK, BAX and BOK can be suppressed and thus, preventing cancer cells to undergo apoptosis during cancer progression [40].

The IAP family (e.g., cIAP-1, Survivin and XIAP) and the Bcl-2 protein are anti-apoptotic proteins that, in response to a variety of pro-apoptotic stimuli, aim to prevent apoptosis in cancer cells, where they can be overexpressed. Their mode of action usually is the caspase inhibition, but regulation of cell division, cell cycle and signaling pathways are influenced, as well [40]. This scenario leads to poor prognosis and is related to an increased tumor recurrence [41]. The downregulation of these proteins can improve pro-apoptotic activity and are promising targets for chemotherapy [40]. The same rationale can be involved in the suppression of pro-apoptotic proteins such as BAX and BAK [42].

### 1.5. Nanosystems

Nanoparticles can be put together to create highly customized nanosystems with a diverse set of features that can help raise specificity to neoplastic cells and safeness to the treatment, along with other advantages such as better solubility and stability. The key design is to turn nanoparticles into complex functionalized carriers of anti-cancer agents, tumor microenvironmental modulators and targeting molecules, delivering them to tumor sites in a highly specific fashion [5]. In fact, a few approved nanomedicines are already available such as Doxil^®^ and Caelyx^®^ which are liposomal nanoparticle formulation that increase the bioavailability of doxorubicin (DOX) to tumor cells, and that can be further functionalized using nanotechnology to overcome other limitations [43].

There are a lot of strategies that can be explored to achieve the aforementioned objectives. Stimuli-response nanomedicines use the tumor microenvironmental features to trigger a specific response such as releasing the nanosystems cargo (e.g., siRNA, chemotherapy agents) when there is a variation in pH [44] or redox state of the cell [45], or in the presence of overexpressed enzymes [36]. These stimuli change the structure of the nanoparticle facilitating the release of the cargo. In addition, more than one stimulus can be exploited [46].

Exogenic triggers such as the near infrared radiation (NIR) which is less invasive can use local heating agents (e.g., graphene nanosheets, magnetic and gold nanoparticles) to sensitize neoplastic cells and to trigger the release of a cargo. This strategy is called photothermal therapy and many formulations have been developed successfully to target cancer cells using this strategy, which is promising [47,48].

There are many molecules (biomarkers) that are present or overexpressed only in neoplastic cells and that can be used as receptors for delivering purposes. For instance, integrins αvβ3 are overexpressed in the tumoral endothelium during angiogenesis, are present in prostate cancer, melanomas, breast carcinomas and glioblastomas [49,50] and can be used as a receptor to the RGD (Arginylglycylaspartic acid) peptide ligand attached to nanosystems which will lead the whole system to bioaccumulate in tumor sites, overcoming cancer resistance [51]. The identification of new biomarkers to be used as targeting receptors is an ongoing process [52].

Nanosystems can deliver imaging and therapeutic molecules to the tumorigenic site in order to improve diagnosis, to assess if an anti-cancer agent is performing successfully and/or to screen new places of tumor sites after treatment or recurrence. When a nanosystem acts at the same time as an imaging and therapeutic agent, it is known as a theranostic [53]. The imaging function can be achieved assembling nanosystems attached to radionuclides such as the technetium-99-metastable and other imaging agents which can give real-time information about the function of the nanosystem in the body [54,55].

The strategies that has been developed and applied for years in nanosystems can be used and coupled with the ones that aim to modulate the ABC efflux function, enhancing the efficacy of today’s formulations (e.g., all generations of ABC inhibitors) that still holds many issues as described before [46]. Graphene [55,56], silica mesoporous [57] and polymeric nanoparticles [58] are promising vehicles that can be built with high specificity to tumor cells, fulfilling these strategies [46].

### 1.6. Graphene

Graphene [56] is an allotrope of carbon. It is a two-dimensional structure organized in a honeycomb configuration, forming an atom-thick sheet, linked together through sp2-sp2 carbon bonds [59]. Graphene has many properties such as strong elasticity, high conductivity, thermal stability. In addition, the structure of graphene allows the binding of other molecules through π–π stacking, which is useful for functionalization [60,61]. Pristine graphene is hydrophobic and so as, needs a biological fluid to make it available for new applications in nanosystems to treat diseases. Graphene oxide, reduced graphene oxide, graphene nanoribbon and graphene quantum dots (GQDs) are a few examples of how graphene can be modified to serve a particular purpose in nanotechnology and biomedical applications [60,61].

Mitoxantrone (MIT) is a well-stablished antineoplastic agent used in the treatment of breast cancer, leukemia and Hodgkin’s lymphoma. However, MIT is a substrate to MDR [62]. Graphene Oxide (GO) displays high drug loading capacity, energy absorption and photothermal properties which can be explored in photothermal therapies to sensitize cancer cells. Hou and collaborators [47] developed a multifunctional hyaluronic acid modified graphene oxide loaded with MIT to overcome the MDR in MIT resistance in breast cancer cell lines, which successfully bypassed the efflux mediated by P-gp transporters, delivering MIT. Silencing the expression of MDR genes using GO is possible as well. Li and collaborators [63] assembled a GO nanoparticle containing two molecular beacons and DOX, which successfully bypassed the resistance to DOX found in MCF-7/ADR breast cell line.

Graphene quantum dots have exceptional physicochemical properties, good biocompatibility, low cytotoxic activity and emit intrinsic fluorescent light [53]. In addition to these properties, it was recently found [64] that a preincubation with GQDs can inhibit P-gp in MCF-7/ADR cell line-which overexpress this ABC transporter-via alternation modulation of the integrity and permeability of the cell membrane or depleting ATP pools. In addition, GQDs seems to be downregulating the P-gp (MDR1), MRP1 and BCRP genes of every cell line investigated in that study via interaction with their C-rich promoter region. This finding is interesting because the GQDs nanoparticle could be coupled with other strategies that aim to modulate the ABC function in a synergistic manner.

### 1.7. Mesoporous Silica Nanoparticles

Mesoporous silica nanoparticles (MSN) are structures composed of silica organized in a well-ordered internal mesopores with large pore volume and surface area, presenting properties such as tunable size and shape, cost-effective fabrication, high drug loading capability, easy surface modification, well-tolerated in vitro and in vivo [45] and already applied in many therapeutic and diagnostic formulations for anticancer activity [65,66].

Zhang and collaborators [67] introduced a polymer-lipid combined layer coated with MSN and loaded with irinotecan (CPT-11), a BCRP inhibitor. This nanosystem is pH sensitive in the acid tumor microenvironment which lead to the depolymerization of the nanosystem, releasing CPT-11 with enhanced uptake by MCF-7/ADR breast cells. This formulation could be used in combination with chemotherapy to treat resistant breast cancer.

### 1.8. Polymeric Nanoparticles

There is a wide range of natural (e.g., chitosan, cellulose) and synthetic (poly(glycolic acid (PGA), poly lactic acid (PLA)) polymeric materials that can be used to create nanosystems [68]. For instance, Poly (lactic-co-glycolic acid) (PLGA) is a synthetic and biodegradable polyester with properties that has increased biocompatibility and low toxicity. In addition, it has already been approved by the Food and Drug Administration (FDA) for medical applications and so as, the investigation of new nanosystems using PLGA has received great attention [69,70]. There already are a few formulations aiming to bypass the MDR in cancer using PLGA [71,72,73].

Downregulating MDR genes and delivering anti-cancer drugs at the same time can be achieved using polymeric nanoparticles [74]. For instance, coformulation of curcumin and doxorubicin in PLGA downregulated the expression of P-gp/MDR1 and BCL-2 helping DOX accumulate in leukemic cells [75]. In another study [76], authors created a porphyrin-polylactice nanoparticle loaded with a P-gp inhibitor and DOX that re-sensitized DOX-resistant breast cells showing that a synergistic combination of a set of features can overcome MDR.

More recently, Takahasi and collaborators [77] developed an anticancer polymer to target dormant cancer cells which are largely resistant to chemotherapy, assembling a methacrylate random copolymer with cationic and hydrophobic side chains to target overexpressed phosphatidylserine. This copolymer would act as an anticancer peptide killing the dormant cells, which was observed in the treatment of prostate cancer cell lines.

## 2. Results

### 2.1. Evaluation of the Cytotoxicity of Nanomaterials

**Figure 1:**
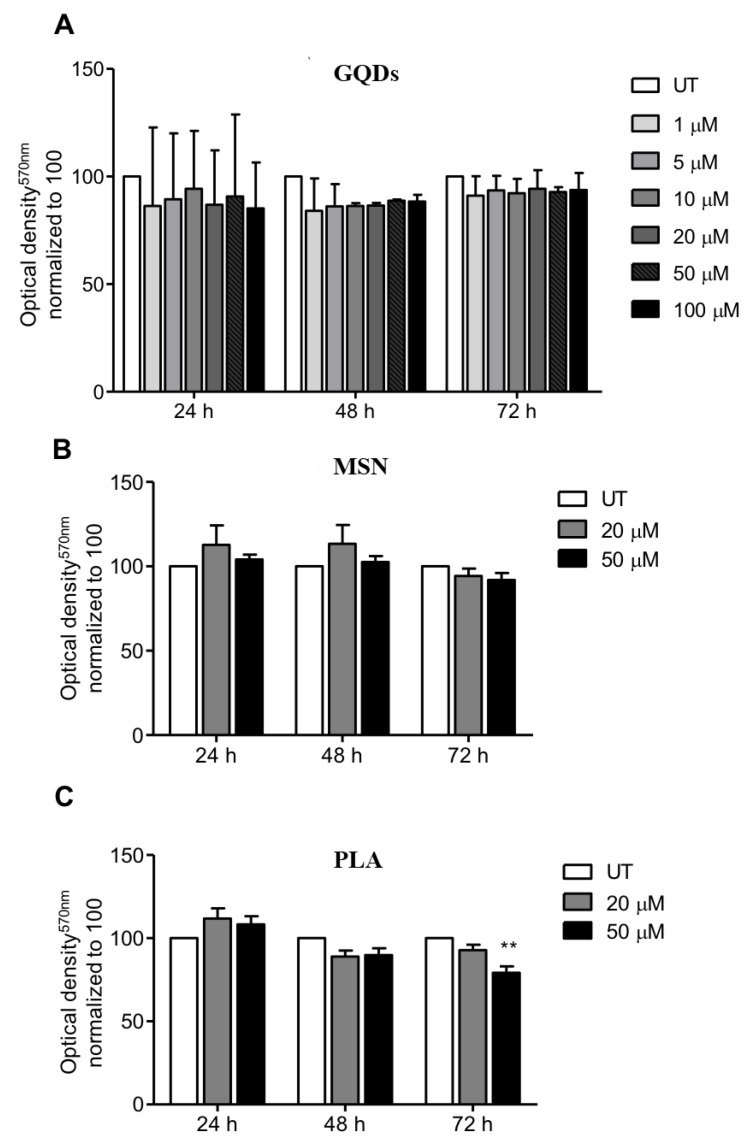
Profile of cell viability of breast cancer cells exposed to a range of nanopolymers concentrations. MDA-MB-231 breast cancer cells were treated with (**A**) graphene quantum dots (GQDs), (**B**) mesoporous silica nanoparticles (MSN) or (**C**) polymeric lactic acid (PLA) for 24, 48 and 72 h. The MTT assay was performed and optical density was obtained at 570 nm. The graphs represent the mean ± standard deviation from three independent experiments. UT: Untreated cells. Statistical significance was analyzed by the one-way ANOVA test (* *p* < 0.05; ** *p* < 0.01).

We first evaluated the effects of three types of nanomaterials (carbon-based GQDs, ceramic-based MSN and PLA nanoparticles) on the viability of MDA-MB-231 cells (human triple negative breast cancer cell line). The selection of the breast cancer cell line MDA-MB-231 was because human breast adenocarcinoma MBA-MD-231 cells express higher levels of pyruvate kinase M2 isoform (PKM2) when compared to MCF-7 cells. This is interesting because PKM2 is the last rate-limiting enzyme in glycolysis, which usually is the main source of energy in cancer cells, giving this cell line an advantage in survivability and thus, more resistance [78]. In case of GQDs nanoparticles, MBA-MD-231 cells were treated with 1 µM, 5 µM, 10 µM, 20 µM, 50 µM and 100 µM for periods of 24, 48 and 72 h and cell viability was determined using the standard MTT cytotoxicity assay. As shown in Figure 1A, treated cells did not exhibit any significant cytotoxicity up to a concentration of 100 µM for all the three treatment incubation durations compared to the untreated cells clearly indicating that GQDs nanoparticles are not cytotoxic to MDA-MB-231 cells. We also performed MTT cytotoxicity assays when MDA-MB-231 cells were treated with 20 µM and 50 µM of mesoporous silica nanoparticles for periods of 24, 48 and 72 h. Similar to GQDs treatments, both tested concentrations of MSN for incubation periods of 24, 48 and 72 h did not show any significant cytotoxicity towards MDA-MB-231 cells compared to untreated cells (Figure 1B). When MDA-MB-231 cells were treated with 20 µM of PLA for periods of 24, 48 and 72 h, again we did not observe any cytotoxicity. However, treatment with 50 µM of PLA for 72 h did show slight decrease in cell viability whereas no cytotoxicity was observed for 24 and 48 h treatment durations.

### 2.2. Colonogenic Assay

**Figure 2:**
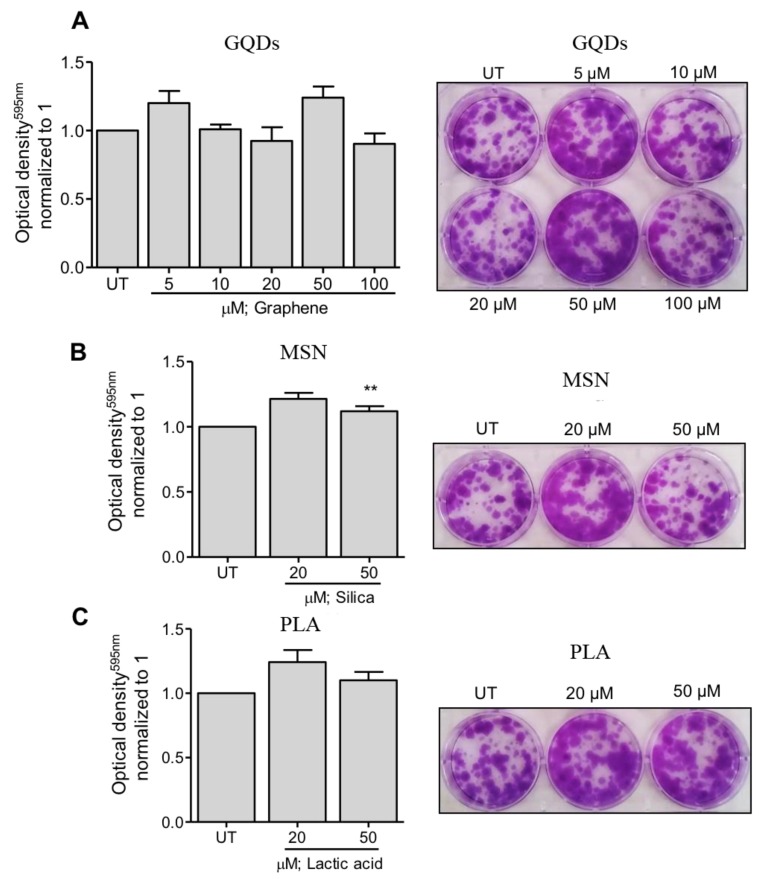
Clonogenicity capacity following treatment of breast cancer cells with a range of (**A**) graphene quantum dots (GQDs), (**B**) mesoporous silica nanoparticles (MSN) or (**C**) polymeric lactic acid (PLA) concentrations. MDA-MB-231 breast cancer cells were treated with nanopolymers for 24 h, after which fresh media was replaced in plates. After colony formation, cells were stained with crystal violet. Colonies were dissolved and optical density was measured at 595 nm. The graphs represent the mean ± standard deviation from three independent experiments. UT: Untreated cells. Statistical significance was analyzed by the Student´s *t* test (* *p* < 0.05; ** *p* < 0.01).

Next, we evaluated the effects of three types of nanomaterials on the colony formation capacity of MDA-MB-231 cells. In case of GQDs nanoparticles, MBA-MD-231 cells were treated with 5 µM, 10 µM, 20 µM, 50 µM and 100 µM and colony formation capacity was determined using the colonogenic assay as described in the Methods section. As shown in Figure 2A, treated cells did not exhibit any significant impairment in the colony formation capacity at any of the tested treatment concentrations. Similar observations were made when colony formation capacity of MBA-MD-231 cells were assessed after treating the cells with 20 µM and 50 µM of MSN or PLA (Figure 2B,C). Together, these results indicate that all the three types of nanoparticles are safe and non-toxic to MBA-MD-231 and do not significantly impact the colony formation capacity of these cells.

### 2.3. Effects of Nanomaterials on P-gp Expression Levels

**Figure 3:**
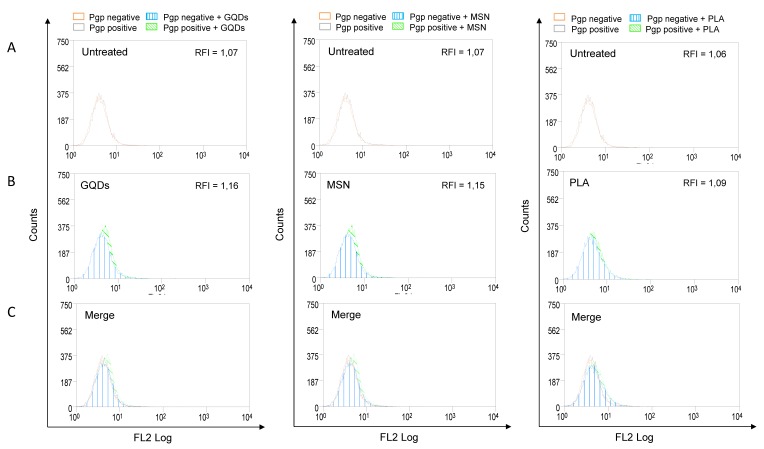
P-glycoprotein (Pgp) expression profile in nanopolymer-treated breast cancer cells. MDA-MB-231 cells were treated with 50 µM graphene quantum dots (GQDs), mesoporous silica nanoparticles (MSN) or polymeric lactic acid (PLA) for 72 h and had P-gp expression compared with untreated cells (**A**,**B**). Cells were stained with PE-conjugated Pgp monoclonal antibody (UIC2, Coulter, USA) and evaluated by flow cytometry. For better visualization of differences between untreated and nanopolymer-treated cells, histograms were merged (**C**). The results were expressed by Relative Fluorescence Intensity (RFI), calculated by the ratio between the fluorescence intensity in cells treated with UIC2 and the fluorescence intensity in cells.

Increased expression of P-gp is frequently associated with the multidrug resistance of various types of cancers. In addition, P-gp can significantly affect the absorption and distribution of many commonly used anticancer agents [12]. Therefore, we also evaluated the effects of three types of nanomaterials on the membrane expression levels of P-gp. MBA-MD-231 cells were treated with 50 µM of GQDs, MSN or PLA for 72 h and the levels of membrane P-gp were determined using flow cytometry-based assay as described in the Methods section. As shown in Figure 3, none of the three nanomaterials under the tested conditions significantly modulated the expression levels of P-gp.

### 2.4. Effects of Nanomaterials on Drug Resistance Related Proteins

**Figure 4:**
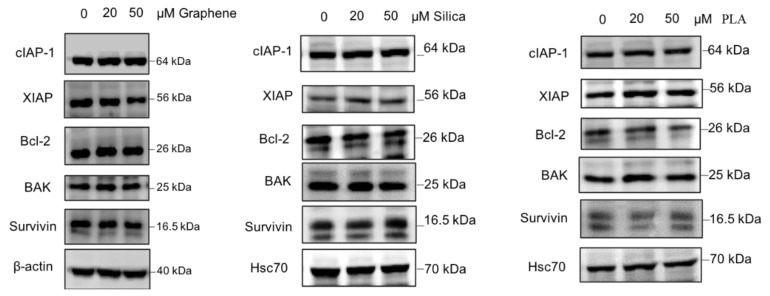
Expression pattern of proteins related to drug resistance in nanopolymer-treated MDA-MB-231 cells. MDA-MB-231 cells were treated with graphene quantum dots (GQDs), mesoporous silica nanoparticles (MSN), polymeric lactic acid (PLA) during 24 h and expression of c-IAP1, XIAP, Bcl-2, BAK and Survivin proteins were analyzed by Western blotting. Hsc70 or β-actin was used as an internal control. The blots are representative of three independent experiments.

Based on the available literature, there are many proteins involved in various cellular pathways that have been associated with cancer drug resistance [40,79]. Therefore, we also evaluated the effects of the different nanomaterials on the expression levels of some proteins with established role in drug resistance. The proteins which were investigated in this study include cIAP-1, XIAP, Bcl-2, BAK and Survivin. For loading control levels of β-actin or Hsc70 were determined as well. MBA-MD-231 cells were treated with 20 µM and 50 µM of GQDs, MSN or PLA for 72 h and the levels of chemoresistance related proteins were determined using immunoblot analysis. As shown in Figure 4, expression levels of most of the proteins including cIAP-1, Bcl-2, BAK and Survivin did not appear to change significantly after treatments with all three types of nanomaterials. However, XIAP protein levels were reduced with 50 µM of GQDs whereas 20 µM and 50 µM treatment of PLA appears to increase the expression levels.

### 2.5. Effects of Nanomaterials on MRP1 Efflux Transporter Protein

**Figure 5:**
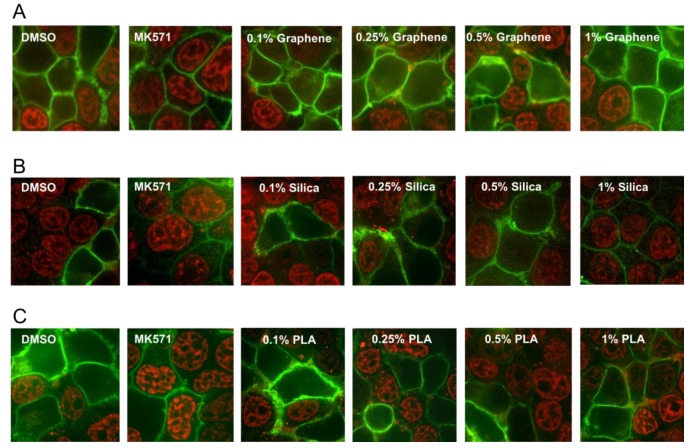
Effect of nanopolymer treatments on MRP1 efflux activity. Doxorubicin accumulation assay was used to measure MRP1 efflux activity. HEK293T cells transiently transfected with MRP1-GFP (green) were pre-treated with 0.1%, 0.25%, 0.5%, 1% Graphene (quantum dots) (**A**), (mesoporous) Silica (**B**), PLA nanoparticles (**C**) or 50 μM of MK571 (known MRP1 inhibitor), before incubation with doxorubicin (red) at 37 °C for 1 h. Images were acquired using confocal microscopy. GFP and doxorubicin were excited at 488 nm, and emission detected at 475/42 and 605/64 nm, respectively.

MRP1 is a membrane transporter protein and can pump out a wide variety of chemical structures including drugs, metals and various toxic compounds and their metabolites. Increased MRP1 activity is also associated with drug resistance and this transporter protein also can significantly affect the absorption and distribution of many cancer drugs [12]. Therefore, we also investigated the effects of the three different types of nanomaterials on the efflux activity of MRP1 transporter. MRP1 transport activity was evaluated by detecting the accumulation of the fluorescent anticancer drug, doxorubicin, a well-known substrate of MRP1. HEK293T (Human embryonic kidney 293 that expresses a mutant version of the SV40 large T antigen) cells were transiently transfected with MRP1-GFP vector and confocal microscopy was used to visualize the effect of various concentrations (0.1%, 0.25%, 0.5% and 1%) of the GQDs, MSN or PLA on MRP1-mediated doxorubicin efflux in live cells. As shown in Figure 5, cells treated with DMSO (vehicle) showed high doxorubicin accumulation in the nuclei of non-transfected cells, while doxorubicin fluorescence was very low or undetectable in cells expressing GFP-tagged MRP1 (MRP1-GFP). MRP1-mediated efflux of doxorubicin was blocked by MK571 treatment (50 µM), a commonly used inhibitor of MRP1 transport activity. If any of the nanoparticles were substrates of MRP1 then they may decrease the efflux of doxorubicin by MRP1 due to competitive inhibition. As shown in Figure 5A, none of the tested concentrations of GQDs demonstrated MRP1 inhibition. These results indicated that GQDs did not interfere with the doxorubicin efflux by MRP1. In contrast, treatment of cells with 1% MSN strongly inhibited doxorubicin efflux by MRP1. We also observed some modest inhibition of doxorubicin efflux by MRP1 in the cells treated with 1% PLA.

## 3. Discussion

Multidrug resistance is a multifactorial biological process that indicates a phenotype in malignant tumor cells in which they are resistant to structurally and functionally unrelated compounds, surviving chemotherapy [14]. MDR can be present on the get go of the first treatment for some tumors (e.g., pancreatic and lung cancer) or can be developed after a few cycles of chemotherapy [18]. There are many mechanisms that lead to multidrug resistance such as: (i) overexpression of transmembrane ABC transporters that efflux drugs from the cytoplasm to the extracellular environment; (ii) defective apoptosis pathways that prevent the cell from undergoing programmed death; (iii) increased DNA damage response pathways that protect the cell from damage induced by agents; (iv) overexpression of metabolism genes involved in detoxification of chemotherapy agents (e.g., cytochrome P450); (v) the tumor microenvironment features that can hinder drug penetration and distribution [80]. Over-expression of ABC drug transporters is the most frequent mechanism of MDR, which is estimated to be responsible for up to 90% of the failures in cancer chemotherapy [46]. Drug transporter proteins such as P-gp and MRP1 are often up-regulated in various types of cancer. 

Among ABC drug transporter proteins, P-gp and MRP1 have a well-established role in MDR. The overexpression of these transporters pumps the chemotherapeutic agents out of cells thereby reducing drug accumulation and causing tumor cells to become resistant [19]. Increased levels of P-gp mRNA and protein have been detected in various types of cancers such as leukemia, kidney, colon, breast and lung cancer [26,81]. The over-expression of P-gp has been linked to poor response to chemotherapy. Although there is considerable overlap in the substrate drugs of MRP1 and P-gp, their tissue expression and physiological substrate profile are significantly different. While substrates for P-gp are mostly hydrophobic, MRP1 can efflux a broad array of physiological organic anions such as leukotriene C_4_ (LTC_4_) and dinitrophenyl-S-glutathione (DNP-SG) [82] and is capable of transporting both conjugated and non-conjugated organic anions while P-gp can transport only non-conjugated drug substrates. Overexpression of MRP1 has been associated with multidrug resistance in lung, breast, prostate cancers and several types of leukemia [19]. In clinics, MRP1 overexpression is a strong prognostic marker in several cancers. For instance, MRP1 overexpression is highly predictive of poor outcome in case of children primary neuroblastoma [83]. 

Many conventional cytotoxic anticancer drugs (e.g., doxorubicin, vincristine and methotrexate) are substrates of ABC drug transporters, which led to a perception that the new targeted therapy drugs which are not very toxic may not be recognized and pumped out by ABC drug transporters. However, recent studies have shown that ABC drug transporters like P-gp and MRP1 can recognize a remarkable variety of these newer targeted therapy drugs and can reduce their efficacy as well [13,19,21,22]. Therefore, ABC drug transporters are considered very important therapeutic targets for the improvement of cancer chemotherapy and to overcome the MDR. A common approach in targeting ABC drug transporter as a strategy for overcoming MDR involves treatment with a combination of an anticancer drug and an ABC transporter inhibitor to increase the efficacy of the anticancer drug. Unfortunately, this approach has not been very successful in clinical trials yet [18].

A relatively new and more promising approach in cancer chemotherapy is the use of various nanosystems for drug delivery to achieve higher specificity and less toxicity [14]. In addition, nanomaterials are also a very powerful tool for tissue imaging for diagnostics and monitoring the progress of treatment [53]. In addition to anticancer drugs, P-gp and MRP1 can also significantly affect the absorption and distribution of a wide variety of drugs, drug-conjugates and various types of endogenous and exogenous chemical structures including nanoparticles. Therefore, it is very important to profile the interaction of various nanomaterials with ABC drug transporters to maximize their positive role. The development of new compounds that aim to modulate the function of ABC proteins is a work in progress [35,36]. New compounds that interact with ABC drug transporters could still induce adverse effects. The main reason is that ABC transporter proteins retain many physiological functions, and compounds can end up targeting and hindering normal tissues [34]. In the light of this issue, a recent strategy to circumvent MDR is to develop an active targeting nanosystem that would reduce or eliminate nonspecific activity in healthy cells and increase penetration in the diseased tissue. These nanosystems can carry old and new anti-cancer compounds to the neoplastic cells avoiding or reducing any possible adverse effect and killing them successfully [46].

In the present study, we analyzed three types of nanomaterials (carbon-based graphene quantum dots, ceramic-based mesoporous silica and polymeric lactic acid nanoparticles) for their potential application to overcome MDR. Using a human triple negative breast cancer cell line, MDA-MB-231, we found that regardless of the concentration, GQDs, MSN and PLA are not cytotoxic to these cells and are safe and harmless (Figure 1). In addition, we also found that irrespective of the concentration, all the three types of nanomaterials did not exhibit any impairment in the colony formation capacity of MDA-MB-231 cells (Figure 2). These results further corroborated the safety of these nanomaterials. 

Increased expression of P-gp is frequently linked with the multidrug resistance of various types of cancers. Various chemical treatments can lead to increased expression of P-gp through transcriptional upregulation. Therefore, we investigated the effects of the nanomaterials on the membrane expression levels of P-gp. Using antibody-based flow cytometry approach we observed that P-gp membrane expression was not significantly altered by any of the three nanomaterials we tested (Figure 3).

There are many proto-onco genes as well as many tumor repressor genes and together they regulate the tumor progression, metastasis and its resistance towards various chemotherapeutic drugs. There are several proteins involved in various pathways that are associated with MDR. The programmed cell death pathway, apoptosis, is one of them and is an important physiological mechanism of proliferation and death control and can be induced by many anticancer drugs. Signaling pathways and anti and pro-apoptotic proteins can be involved in apoptosis and in cancer resistance, as well. Thus, they are interesting targets for chemotherapy to circumvent cancer resistance [40]. Consequently, we investigated the impact of nanomaterial treatments on the expression levels of cIAP-1, XIAP, Bcl-2, BAK and Survivin. None of the three nanomaterials significantly altered the expression of any proteins tested except XIAP protein. Levels of the XIAP protein were reduced by GQDs nanoparticles whereas treatment with PLA increased the protein expression levels confirmed by immunoblotting method. 

MRP1 can significantly affect the bioavailability of a remarkable variety of chemical structures and increased MRP1 activity is also linked with MDR, therefore we investigated the effects of nanomaterials on the transport activity of this transporter. For successful cancer treatment, it is desirable to either inhibit this transporter’s activity in cancer patient or use those agents that are not substrates of this protein. Using doxorubicin accumulation assay as reporter of MRP1 activity, our results indicate that GQDs nanoparticles did not interfere with the MRP1 efflux activity of doxorubicin suggesting it did not compete with doxorubicin and that GQDs nanoparticles would be very suitable for doxorubicin delivery to cancer cells and to bypass the efflux activity of MRP1. In addition, this result also suggests that GQDs nanoparticles would be suitable for the delivery of other MRP1 substrate drugs that bind the transporter at the same binding pocket. In contrast, we observed that 1% MSN can strongly inhibit doxorubicin efflux by MRP1. This result suggests that mesoporous silica nanoparticles are promising nanomaterials in the treatment of tumors where MRP1 overexpression is the main contributor of MDR and that MSN can be combined with MRP1 substrate drugs to improve the drug accumulation within the cells by inhibiting the efflux activity of MRP1. PLA at 1% concentration exhibited moderate inhibition of doxorubicin efflux by MRP1 suggesting this nanomaterial can also be useful to treat MDR due to MRP1 overexpression.

## 4. Methods

### 4.1. Cell Culture

The MDA-MB-231 human breast cancer cell line (invasive breast ductal carcinoma, mutant *TP53*, triple negative) was cultured in Dulbecco’s modified Eagle’s medium supplemented with 10% fetal bovine serum, 100 U/mL penicillin/streptomycin and 2 mM glutamine and maintained in a humidified incubator with 5% CO_2_ at 37 °C. The cell line was authenticated by genotyping of short tandem repeats and monitored for mycoplasma contamination. Cells in exponential growth were exposed to different concentrations of graphene, silica and lactic acid and harvested for cytotoxicity assays as described below.

### 4.2. MTT Assay

In order to evaluate if the different types of nanoparticles under investigation could interfere with cell survival, MTT test in MDA-MB-231 cells was performed. To test the effect of nanoparticles, MDA-MB-231 cells at concentration of 5 × 10^3^ cells were plated on 96 wells plate and incubated with 10% FBS. Then the cells were treated with 1 µM, 5 µM, 10 µM, 20 µM, 50 µM and 100 µM of nanoparticles for periods of 24, 48 and 72h at 37 °C in a humidified atmosphere with 5% CO_2_. After the incubation period, the culture medium was removed and cells were incubated with MTT solution at 1 mg/mL for 2 h. Thereafter, the MTT solution was removed and 200 µL of absolute isopropanol was added to lyse the cells and solubilize the MTT crystals in all conditions. The absorbance of the extract was read at 570 nm using a microplate reader.

### 4.3. Clonogenic Assay

For assessment of colony formation capacity, a total of 2000 MDA-MB-231 cells were seeded into six-well plates and left overnight for adherence, after which they were treated with 20 and 50 µg mesoporous silica (MSN) or polymeric lactic acid (PLA) nanoparticles, as well as increasing concentrations of graphene quantum dots (GQDs). Following 24 h of treatment, cells were cultured in fresh drug-free media and grown for around 14 days until colony formation. Colonies were then washed 3 times with phosphate buffer saline (PBS) and fixed with 100% ethanol for 15 min at room temperature. After three additional washes with PBS, colonies were stained with 0.5% crystal violet for 1 h, washed with flowing water and air-dried. Then, 1 mL of 33% acetic acid was added to each well for crystal violet solubilization. Optical density was measured at 595 nm using an ELISA reader (DTX 800 Multimode Detector from Beckman Coulter, Fullerton, CA, USA). Four independent experiments were performed, and the optical density values obtained in untreated cells were normalized to 1, for comparison across experiments.

### 4.4. Assessment of P-Glycoprotein (P-gp) Expression by Flow Cytometry

To compare P-gp membrane expression in untreated and nanomaterial-treated MDA-MB-231 cells, a total of 5 × 10^5^ cells were stained with PE-conjugated monoclonal antibody against P-gp (clone UIC2, Coulter, USA) and analyzed for flow cytometry. Briefly, cells were treated with 50 µM GQDs, MSN or PLA for 72 h, when they were harvested by trypsinization (0.125% trypsin; Invitrogen) and incubated with 1% bovine serum albumin (BSA) diluted in phosphate buffered saline (PBS) for 15 min. Direct staining was performed with 1 µL P-gp antibody for 30 min at room temperature, after which cells were washed twice with PBS and acquired by flow cytometry (Cyan ADP, Coulter, IN, USA). Results were analyzed using the Summit v4.3 software (Coulter, IN, USA) and expressed as the ratio of fluorescence intensity (RFI), obtained following division of the mean fluorescence of P-gp-stained cells by the mean fluorescence of cells with no P-gp antibody [79].

### 4.5. Western Blotting

For analysis of chemoresistance-related proteins, cells were exposed to 50 µM GQDs, MSN or PLA for 72 h and harvested by trypsinization (0.125% trypsin; Invitrogen). After three washes with PBS, a total of 2 × 10^6^ cells were lysed in Cell Extraction Buffer (Invitrogen, Carlsbad, CA, USA) and quantified using the Bio-Rad protein assay solution (Life Science Research, Hercules, CA, USA). A total of 20 µg proteins were subjected to 10% polyacrylamide gels and Western blotting was performed, as previously described [79]. Antibodies for XIAP (1:1000 dilution; Cell Signaling, MA, USA), Survivin (1:1000 dilution; Cell Signaling, MA, USA), cIAP-1 (1:1000 dilution; Cell Signaling,MA, USA), Bcl-2 (1:1000 dilution; DakoCytomation Denmark A/S, Produktionsvej Glostrup) and Bak (1:1000 dilution; Cell Signaling, MA, USA) were used. As a loading control, membranes were probed with the β-actin antibody (1:1000 dilution; Sigma-Aldrich, MO, USA). Mouse and rabbit secondary antibodies were purchased from. Primary antibodies were detected using horseradish peroxidase linked anti-rabbit (1:40,000 dilution; GE Healthcare^®^, WI, USA) and anti-mouse (1:40000 dilution; GE Healthcare^®,^ WI, USA) conjugates and visualized using the Clarity Max™ detection kit (BioRad Laboratories, Hercules, CA, USA). Bands were detected using a Li-Cor imager (Biociences, Lincoln, NE, USA).

### 4.6. MRP1 Efflux Transporter Protein Activity

Efflux transport activity of MRP1 was measured using doxorubicin accumulation assay. This assay was performed using confocal microscopy. Sterilized cover glasses were placed in 6-well plate and covered with 0.1 mg/mL of poly-D-lysine for 10 min prior to washing with phosphate buffered saline (PBS). HEK293T cells were plated on poly-d-lysine-coated cover glass at a density of 5 × 10^5^ cells/well in 2 mL culture medium. Cells were transiently transfected with 2 µg of an MRP1-GFP expression vector after 24 h using 4 µg of jetPRIME Transfection Reagent with 200 µL of Transfection buffer (Polyplus-transfection SA, Illkirch, France). After 48 h, cells were pre-treated with 0.1%, 0.25%, 0.5% and 1% of 2 mg/mL GQDs, MSN or PLA nanoparticles for 30 min, before incubation with doxorubicin (10 µM) for 1 h. Cells were maintained in buffer (4.5% glucose, 10mM HEPES, PBS containing Ca^2+^ and Mg^2+^) as intracellular fluorescence was visualized using a iMIC digital microscope (TILL Photonics GmbH, Gräfelfing, Germany) equipped with a 1.35 numerical aperture 60× oil-immersion objective. Excitation was done at 488 nm for GFP and doxorubicin, with emission bands of 475/42 and 605/64 nm, respectively. Images were processed using ImageJ (NIH, Bethesda, MD, USA).

### 4.7. Statistical Analysis

Statistical analysis of data was performed using the Graph-Pad Prism 5.0 software (GraphPad Software, San Diego, CA, USA). Differences between means from two groups were compared by the one-way ANOVA test and confirmed by Bonferroni post-test. The values of * *p* < 0.05, ** *p* < 0.01 and *** *p* < 0.005 were considered statistically significant.
